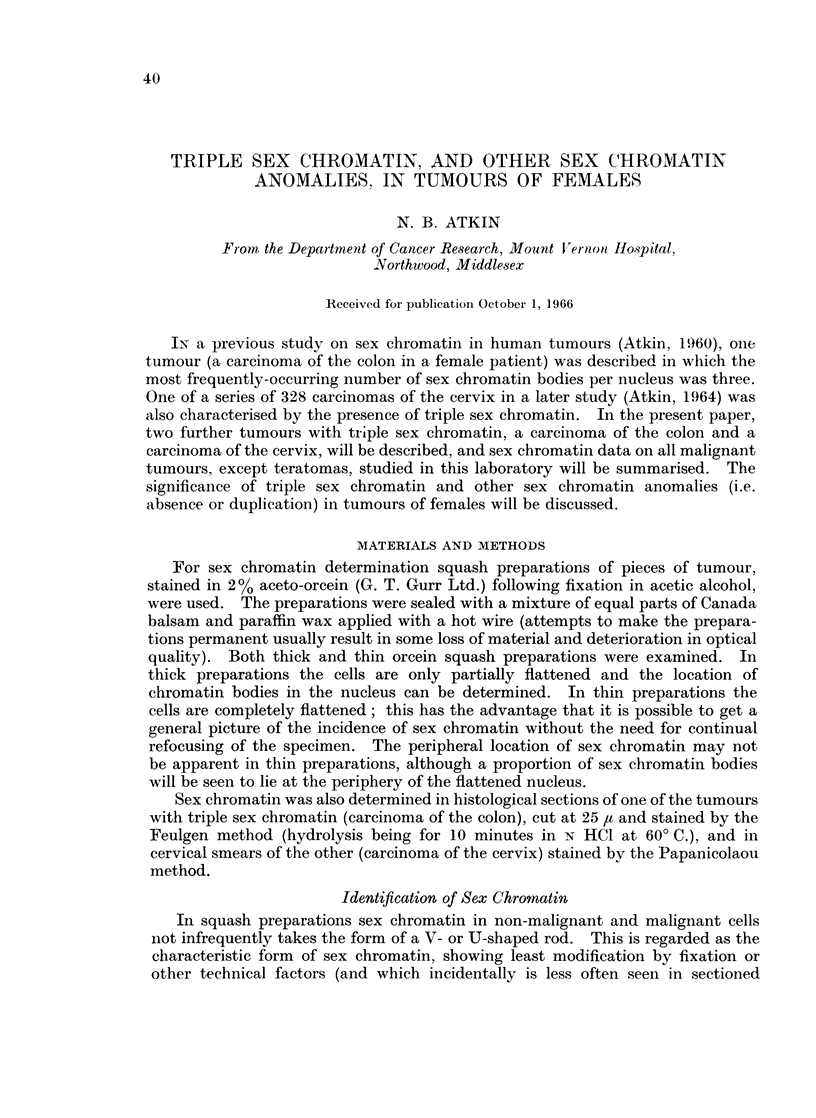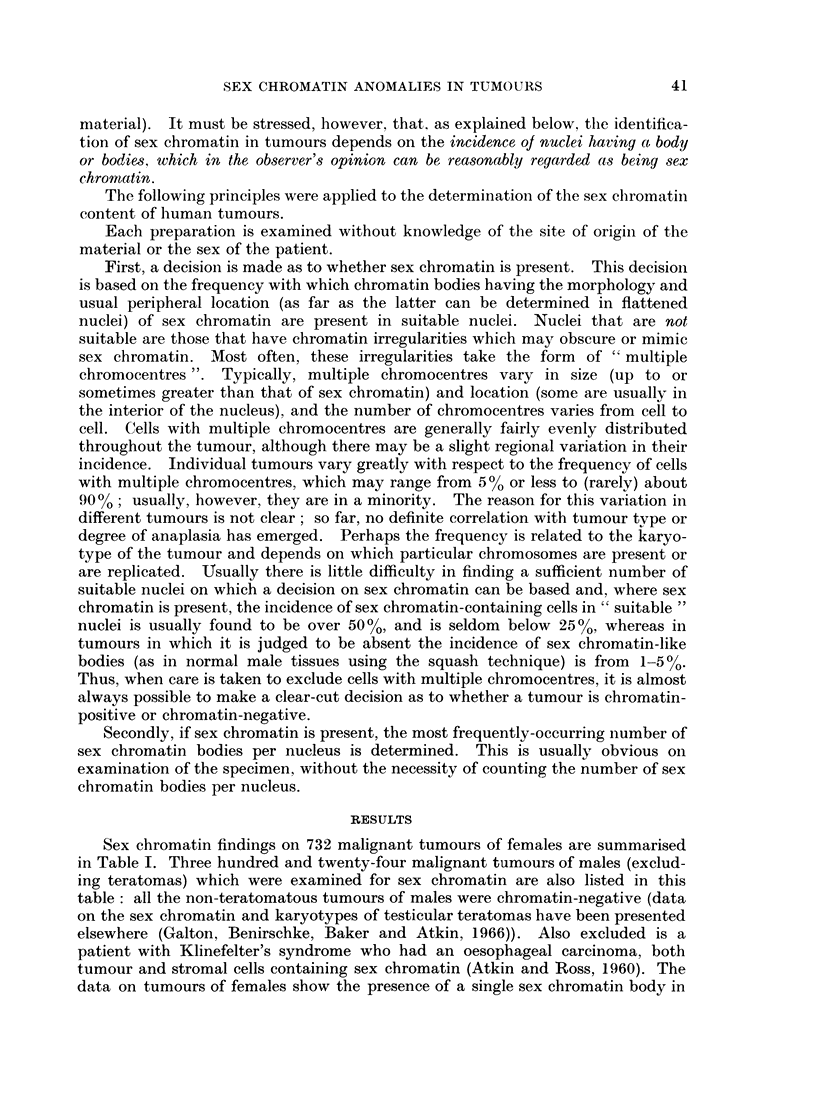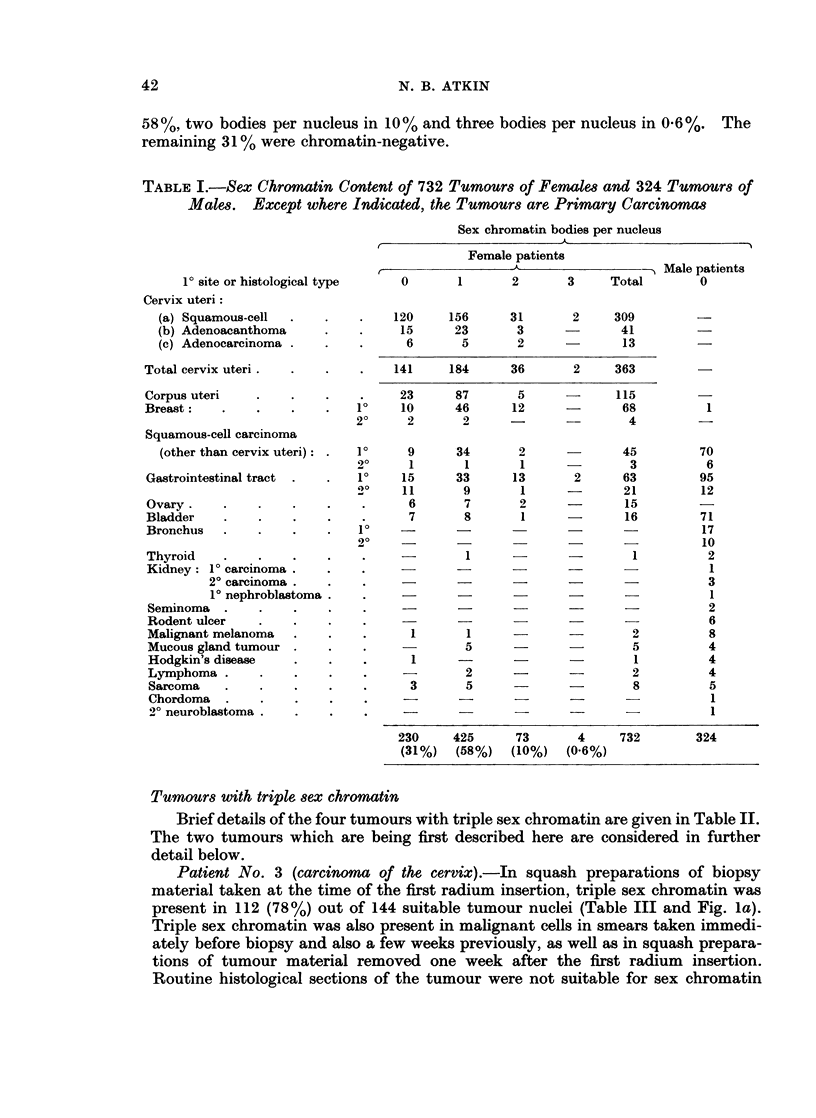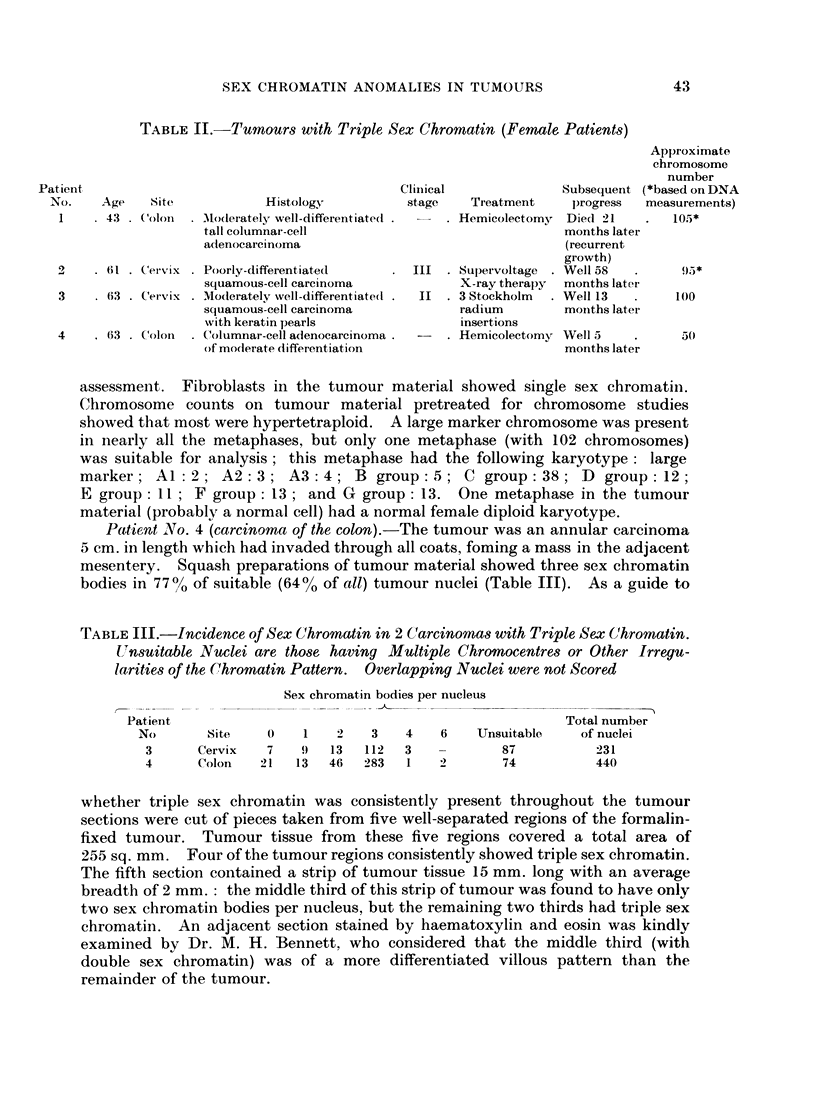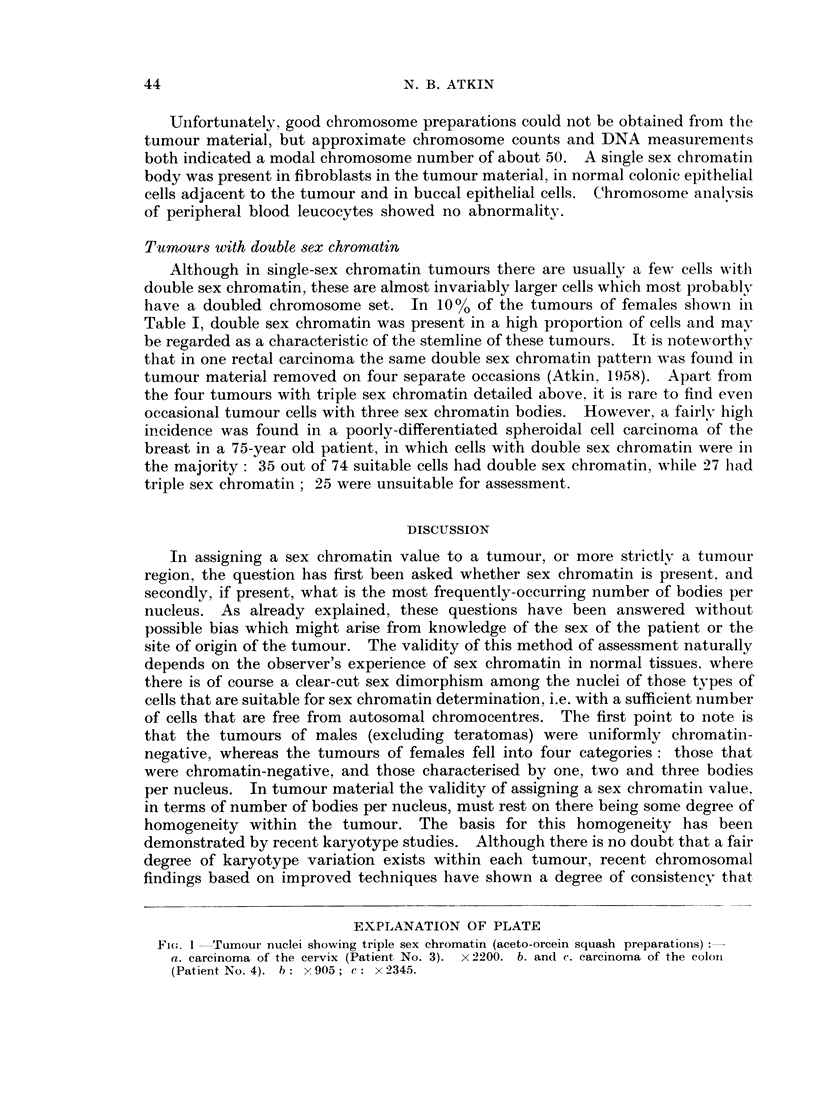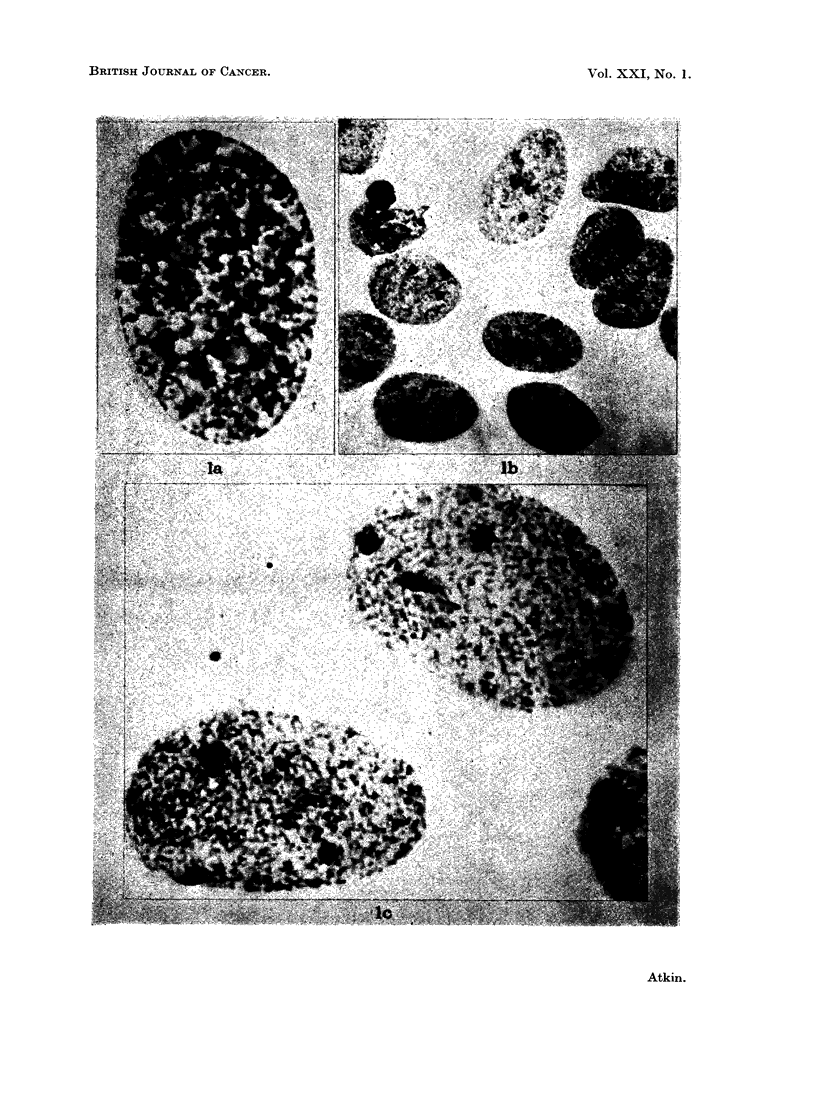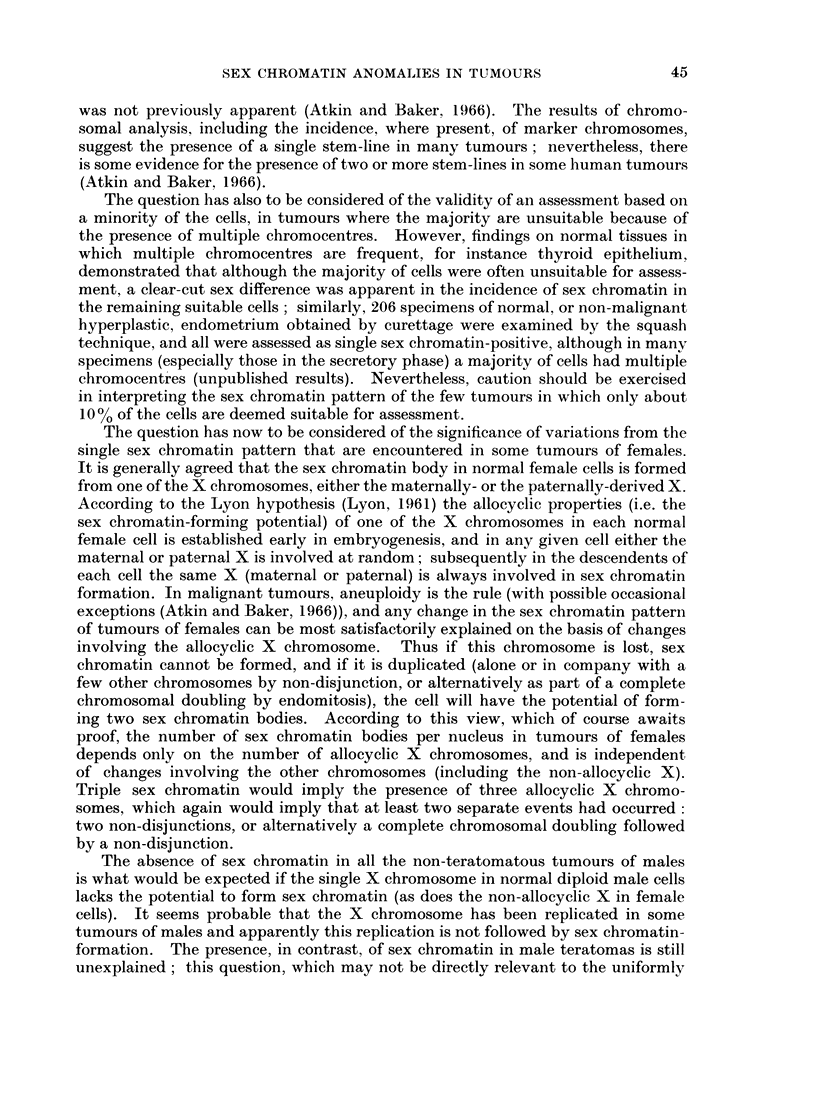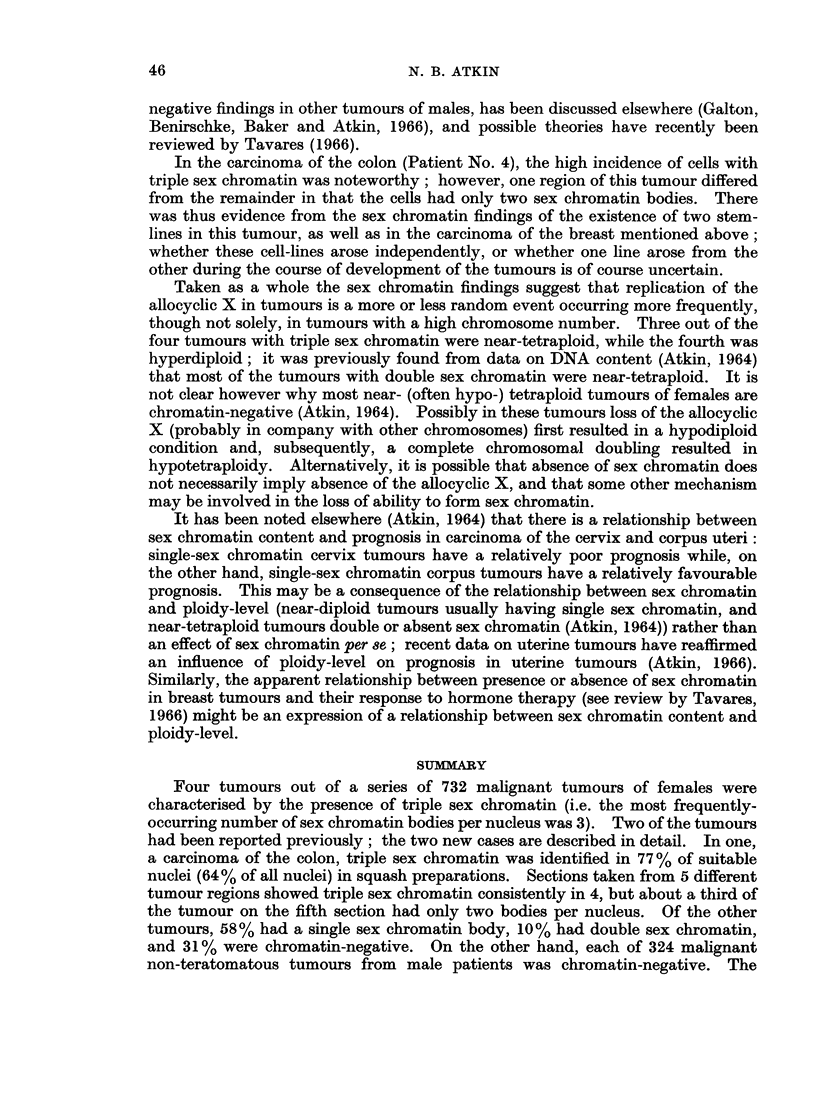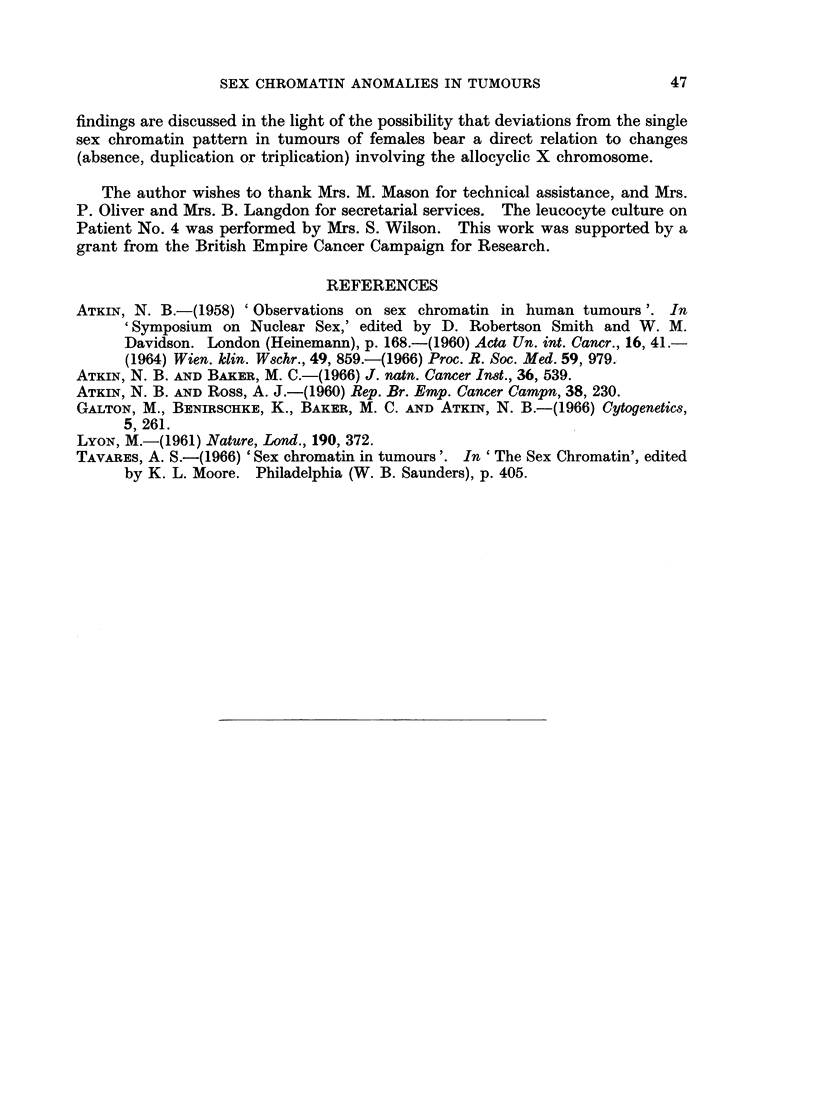# Triple sex chromatin, and other sex chromatin anomalies, in tumours of females.

**DOI:** 10.1038/bjc.1967.6

**Published:** 1967-03

**Authors:** N. B. Atkin

## Abstract

**Images:**


### TRIPLE SEX CHROMATIN, AND OTHER SEX CHROMATIN ANOMALIES, IN TUMOURS OF FEMALES

N. B. ATKIN

*From the Department of Cancer Research, Mount Vernon Hospital, Northwood, Middlesex*

Received for publication October 1, 1966

In a previous study on sex chromatin in human tumours (Atkin, 1960), one tumour (a carcinoma of the colon in a female patient) was described in which the most frequently-occurring number of sex chromatin bodies per nucleus was three. One of a series of 328 carcinomas of the cervix in a later study (Atkin, 1964) was also characterised by the presence of triple sex chromatin. In the present paper, two further tumours with triple sex chromatin, a carcinoma of the colon and a carcinoma of the cervix, will be described, and sex chromatin data on all malignant tumours, except teratomas, studied in this laboratory will be summarised. The significance of triple sex chromatin and other sex chromatin anomalies (i.e. absence or duplication) in tumours of females will be discussed.

### MATERIALS AND METHODS

For sex chromatin determination squash preparations of pieces of tumour, stained in 2% aceto-orcein (G. T. Gurr Ltd.) following fixation in acetic alcohol, were used. The preparations were sealed with a mixture of equal parts of Canada balsam and paraffin wax applied with a hot wire (attempts to make the preparations permanent usually result in some loss of material and deterioration in optical quality). Both thick and thin orcein squash preparations were examined. In thick preparations the cells are only partially flattened and the location of chromatin bodies in the nucleus can be determined. In thin preparations the cells are completely flattened ; this has the advantage that it is possible to get a general picture of the incidence of sex chromatin without the need for continual refocusing of the specimen. The peripheral location of sex chromatin may not be apparent in thin preparations, although a proportion of sex chromatin bodies will be seen to lie at the periphery of the flattened nucleus.

Sex chromatin was also determined in histological sections of one of the tumours with triple sex chromatin (carcinoma of the colon), cut at 25 $\mu$ and stained by the Feulgen method (hydrolysis being for 10 minutes in N HCl at 60° C.), and in cervical smears of the other (carcinoma of the cervix) stained by the Papanicolaou method.

### *Identification of Sex Chromatin*

In squash preparations sex chromatin in non-malignant and malignant cells not infrequently takes the form of a V- or U-shaped rod. This is regarded as the characteristic form of sex chromatin, showing least modification by fixation or other technical factors (and which incidentally is less often seen in sectioned material). It must be stressed, however, that, as explained below, the identification of sex chromatin in tumours depends on the *incidence of nuclei having a body or bodies, which in the observer's opinion can be reasonably regarded as being sex chromatin.*

The following principles were applied to the determination of the sex chromatin content of human tumours.

Each preparation is examined without knowledge of the site of origin of the material or the sex of the patient.

First, a decision is made as to whether sex chromatin is present. This decision is based on the frequency with which chromatin bodies having the morphology and usual peripheral location (as far as the latter can be determined in flattened nuclei) of sex chromatin are present in suitable nuclei. Nuclei that are *not* suitable are those that have chromatin irregularities which may obscure or mimic sex chromatin. Most often, these irregularities take the form of "multiple chromocentres". Typically, multiple chromocentres vary in size (up to or sometimes greater than that of sex chromatin) and location (some are usually in the interior of the nucleus), and the number of chromocentres varies from cell to cell. Cells with multiple chromocentres are generally fairly evenly distributed throughout the tumour, although there may be a slight regional variation in their incidence. Individual tumours vary greatly with respect to the frequency of cells with multiple chromocentres, which may range from 5% or less to (rarely) about 90%; usually, however, they are in a minority. The reason for this variation in different tumours is not clear; so far, no definite correlation with tumour type or degree of anaplasia has emerged. Perhaps the frequency is related to the karyotype of the tumour and depends on which particular chromosomes are present or are replicated. Usually there is little difficulty in finding a sufficient number of suitable nuclei on which a decision on sex chromatin can be based and, where sex chromatin is present, the incidence of sex chromatin-containing cells in "suitable" nuclei is usually found to be over 50%, and is seldom below 25%, whereas in tumours in which it is judged to be absent the incidence of sex chromatin-like bodies (as in normal male tissues using the squash technique) is from 1–5%. Thus, when care is taken to exclude cells with multiple chromocentres, it is almost always possible to make a clear-cut decision as to whether a tumour is chromatin-positive or chromatin-negative.

Secondly, if sex chromatin is present, the most frequently-occurring number of sex chromatin bodies per nucleus is determined. This is usually obvious on examination of the specimen, without the necessity of counting the number of sex chromatin bodies per nucleus.

### RESULTS

Sex chromatin findings on 732 malignant tumours of females are summarised in Table I. Three hundred and twenty-four malignant tumours of males (excluding teratomas) which were examined for sex chromatin are also listed in this table: all the non-teratomatous tumours of males were chromatin-negative (data on the sex chromatin and karyotypes of testicular teratomas have been presented elsewhere (Galton, Benirschke, Baker and Atkin, 1966)). Also excluded is a patient with Klinefelter's syndrome who had an oesophageal carcinoma, both tumour and stromal cells containing sex chromatin (Atkin and Ross, 1960). The data on tumours of females show the presence of a single sex chromatin body in

58%, two bodies per nucleus in 10% and three bodies per nucleus in 0·6%. The remaining 31% were chromatin-negative.

TABLE I.—*Sex Chromatin Content of* 732 *Tumours of Females and* 324 *Tumours of Males. Except where Indicated, the Tumours are Primary Carcinomas*

| | | Sex chromatin bodies per nucleus | | | | | |
|---|---|---|---|---|---|---|---|
| | | Female patients | | | | | Male patients |
| 1° site or histological type | | 0 | 1 | 2 | 3 | Total | 0 |
| Cervix uteri : | | | | | | | |
| (a) Squamous-cell | | 120 | 156 | 31 | 2 | 309 | — |
| (b) Adenoacanthoma | | 15 | 23 | 3 | — | 41 | — |
| (c) Adenocarcinoma | | 6 | 5 | 2 | — | 13 | — |
| Total cervix uteri | | 141 | 184 | 36 | 2 | 363 | — |
| Corpus uteri | | 23 | 87 | 5 | — | 115 | — |
| Breast : | 1° | 10 | 46 | 12 | — | 68 | 1 |
| | 2° | 2 | 2 | — | — | 4 | — |
| Squamous-cell carcinoma (other than cervix uteri) : | 1° | 9 | 34 | 2 | — | 45 | 70 |
| | 2° | 1 | 1 | 1 | — | 3 | 6 |
| Gastrointestinal tract | 1° | 15 | 33 | 13 | 2 | 63 | 95 |
| | 2° | 11 | 9 | 1 | — | 21 | 12 |
| Ovary | | 6 | 7 | 2 | — | 15 | — |
| Bladder | | 7 | 8 | 1 | — | 16 | 71 |
| Bronchus | 1° | — | — | — | — | — | 17 |
| | 2° | — | — | — | — | — | 10 |
| Thyroid | | — | 1 | — | — | 1 | 2 |
| Kidney : 1° carcinoma | | — | — | — | — | — | 1 |
| 2° carcinoma | | — | — | — | — | — | 3 |
| 1° nephroblastoma | | — | — | — | — | — | 1 |
| Seminoma | | — | — | — | — | — | 2 |
| Rodent ulcer | | — | — | — | — | — | 6 |
| Malignant melanoma | | 1 | 1 | — | — | 2 | 8 |
| Mucous gland tumour | | — | 5 | — | — | 5 | 4 |
| Hodgkin's disease | | 1 | — | — | — | 1 | 4 |
| Lymphoma | | — | 2 | — | — | 2 | 4 |
| Sarcoma | | 3 | 5 | — | — | 8 | 5 |
| Chordoma | | — | — | — | — | — | 1 |
| 2° neuroblastoma | | — | — | — | — | — | 1 |
| | | 230 (31%) | 425 (58%) | 73 (10%) | 4 (0·6%) | 732 | 324 |

*Tumours with triple sex chromatin*

Brief details of the four tumours with triple sex chromatin are given in Table II. The two tumours which are being first described here are considered in further detail below.

*Patient No.* 3 (*carcinoma of the cervix*).—In squash preparations of biopsy material taken at the time of the first radium insertion, triple sex chromatin was present in 112 (78%) out of 144 suitable tumour nuclei (Table III and Fig. 1*a*). Triple sex chromatin was also present in malignant cells in smears taken immediately before biopsy and also a few weeks previously, as well as in squash preparations of tumour material removed one week after the first radium insertion. Routine histological sections of the tumour were not suitable for sex chromatin

TABLE II.—*Tumours with Triple Sex Chromatin (Female Patients)*

| Patient No. | Age | Site | Histology | Clinical stage | Treatment | Subsequent progress | Approximate chromosome number (*based on DNA measurements) |
|---|---|---|---|---|---|---|---|
| 1 | 43 | Colon | Moderately well-differentiated tall columnar-cell adenocarcinoma | — | Hemicolectomy | Died 21 months later (recurrent growth) | 105* |
| 2 | 61 | Cervix | Poorly-differentiated squamous-cell carcinoma | III | Supervoltage X-ray therapy | Well 58 months later | 95* |
| 3 | 63 | Cervix | Moderately well-differentiated squamous-cell carcinoma with keratin pearls | II | 3 Stockholm radium insertions | Well 13 months later | 100 |
| 4 | 63 | Colon | Columnar-cell adenocarcinoma of moderate differentiation | — | Hemicolectomy | Well 5 months later | 50 |

assessment. Fibroblasts in the tumour material showed single sex chromatin. Chromosome counts on tumour material pretreated for chromosome studies showed that most were hypertetraploid. A large marker chromosome was present in nearly all the metaphases, but only one metaphase (with 102 chromosomes) was suitable for analysis ; this metaphase had the following karyotype : large marker ; A1 : 2 ; A2 : 3 ; A3 : 4 ; B group : 5 ; C group : 38 ; D group : 12 ; E group : 11 ; F group : 13 ; and G group : 13. One metaphase in the tumour material (probably a normal cell) had a normal female diploid karyotype.

*Patient No.* 4 (*carcinoma of the colon*).—The tumour was an annular carcinoma 5 cm. in length which had invaded through all coats, foming a mass in the adjacent mesentery. Squash preparations of tumour material showed three sex chromatin bodies in 77% of suitable (64% of *all*) tumour nuclei (Table III). As a guide to

TABLE III.—*Incidence of Sex Chromatin in 2 Carcinomas with Triple Sex Chromatin. Unsuitable Nuclei are those having Multiple Chromocentres or Other Irregularities of the Chromatin Pattern. Overlapping Nuclei were not Scored*

| Patient No | Site | Sex chromatin bodies per nucleus: 0 | 1 | 2 | 3 | 4 | 6 | Unsuitable | Total number of nuclei |
|---|---|---|---|---|---|---|---|---|---|
| 3 | Cervix | 7 | 9 | 13 | 112 | 3 | – | 87 | 231 |
| 4 | Colon | 21 | 13 | 46 | 283 | 1 | 2 | 74 | 440 |

whether triple sex chromatin was consistently present throughout the tumour sections were cut of pieces taken from five well-separated regions of the formalin-fixed tumour. Tumour tissue from these five regions covered a total area of 255 sq. mm. Four of the tumour regions consistently showed triple sex chromatin. The fifth section contained a strip of tumour tissue 15 mm. long with an average breadth of 2 mm. : the middle third of this strip of tumour was found to have only two sex chromatin bodies per nucleus, but the remaining two thirds had triple sex chromatin. An adjacent section stained by haematoxylin and eosin was kindly examined by Dr. M. H. Bennett, who considered that the middle third (with double sex chromatin) was of a more differentiated villous pattern than the remainder of the tumour.

Unfortunately, good chromosome preparations could not be obtained from the tumour material, but approximate chromosome counts and DNA measurements both indicated a modal chromosome number of about 50. A single sex chromatin body was present in fibroblasts in the tumour material, in normal colonic epithelial cells adjacent to the tumour and in buccal epithelial cells. Chromosome analysis of peripheral blood leucocytes showed no abnormality.

*Tumours with double sex chromatin*

Although in single-sex chromatin tumours there are usually a few cells with double sex chromatin, these are almost invariably larger cells which most probably have a doubled chromosome set. In 10% of the tumours of females shown in Table I, double sex chromatin was present in a high proportion of cells and may be regarded as a characteristic of the stemline of these tumours. It is noteworthy that in one rectal carcinoma the same double sex chromatin pattern was found in tumour material removed on four separate occasions (Atkin, 1958). Apart from the four tumours with triple sex chromatin detailed above, it is rare to find even occasional tumour cells with three sex chromatin bodies. However, a fairly high incidence was found in a poorly-differentiated spheroidal cell carcinoma of the breast in a 75-year old patient, in which cells with double sex chromatin were in the majority : 35 out of 74 suitable cells had double sex chromatin, while 27 had triple sex chromatin ; 25 were unsuitable for assessment.

### DISCUSSION

In assigning a sex chromatin value to a tumour, or more strictly a tumour region, the question has first been asked whether sex chromatin is present, and secondly, if present, what is the most frequently-occurring number of bodies per nucleus. As already explained, these questions have been answered without possible bias which might arise from knowledge of the sex of the patient or the site of origin of the tumour. The validity of this method of assessment naturally depends on the observer's experience of sex chromatin in normal tissues, where there is of course a clear-cut sex dimorphism among the nuclei of those types of cells that are suitable for sex chromatin determination, i.e. with a sufficient number of cells that are free from autosomal chromocentres. The first point to note is that the tumours of males (excluding teratomas) were uniformly chromatin-negative, whereas the tumours of females fell into four categories : those that were chromatin-negative, and those characterised by one, two and three bodies per nucleus. In tumour material the validity of assigning a sex chromatin value, in terms of number of bodies per nucleus, must rest on there being some degree of homogeneity within the tumour. The basis for this homogeneity has been demonstrated by recent karyotype studies. Although there is no doubt that a fair degree of karyotype variation exists within each tumour, recent chromosomal findings based on improved techniques have shown a degree of consistency that

---

EXPLANATION OF PLATE

FIG. 1.—Tumour nuclei showing triple sex chromatin (aceto-orcein squash preparations) :— *a*. carcinoma of the cervix (Patient No. 3). ×2200. *b*. and *c*. carcinoma of the colon (Patient No. 4). *b* : ×905 ; *c* : ×2345.

1a 1b 1c

Atkin.

was not previously apparent (Atkin and Baker, 1966). The results of chromosomal analysis, including the incidence, where present, of marker chromosomes, suggest the presence of a single stem-line in many tumours ; nevertheless, there is some evidence for the presence of two or more stem-lines in some human tumours (Atkin and Baker, 1966).

The question has also to be considered of the validity of an assessment based on a minority of the cells, in tumours where the majority are unsuitable because of the presence of multiple chromocentres. However, findings on normal tissues in which multiple chromocentres are frequent, for instance thyroid epithelium, demonstrated that although the majority of cells were often unsuitable for assessment, a clear-cut sex difference was apparent in the incidence of sex chromatin in the remaining suitable cells ; similarly, 206 specimens of normal, or non-malignant hyperplastic, endometrium obtained by curettage were examined by the squash technique, and all were assessed as single sex chromatin-positive, although in many specimens (especially those in the secretory phase) a majority of cells had multiple chromocentres (unpublished results). Nevertheless, caution should be exercised in interpreting the sex chromatin pattern of the few tumours in which only about 10% of the cells are deemed suitable for assessment.

The question has now to be considered of the significance of variations from the single sex chromatin pattern that are encountered in some tumours of females. It is generally agreed that the sex chromatin body in normal female cells is formed from one of the X chromosomes, either the maternally- or the paternally-derived X. According to the Lyon hypothesis (Lyon, 1961) the allocyclic properties (i.e. the sex chromatin-forming potential) of one of the X chromosomes in each normal female cell is established early in embryogenesis, and in any given cell either the maternal or paternal X is involved at random ; subsequently in the descendents of each cell the same X (maternal or paternal) is always involved in sex chromatin formation. In malignant tumours, aneuploidy is the rule (with possible occasional exceptions (Atkin and Baker, 1966)), and any change in the sex chromatin pattern of tumours of females can be most satisfactorily explained on the basis of changes involving the allocyclic X chromosome. Thus if this chromosome is lost, sex chromatin cannot be formed, and if it is duplicated (alone or in company with a few other chromosomes by non-disjunction, or alternatively as part of a complete chromosomal doubling by endomitosis), the cell will have the potential of forming two sex chromatin bodies. According to this view, which of course awaits proof, the number of sex chromatin bodies per nucleus in tumours of females depends only on the number of allocyclic X chromosomes, and is independent of changes involving the other chromosomes (including the non-allocyclic X). Triple sex chromatin would imply the presence of three allocyclic X chromosomes, which again would imply that at least two separate events had occurred : two non-disjunctions, or alternatively a complete chromosomal doubling followed by a non-disjunction.

The absence of sex chromatin in all the non-teratomatous tumours of males is what would be expected if the single X chromosome in normal diploid male cells lacks the potential to form sex chromatin (as does the non-allocyclic X in female cells). It seems probable that the X chromosome has been replicated in some tumours of males and apparently this replication is not followed by sex chromatin-formation. The presence, in contrast, of sex chromatin in male teratomas is still unexplained ; this question, which may not be directly relevant to the uniformly negative findings in other tumours of males, has been discussed elsewhere (Galton, Benirschke, Baker and Atkin, 1966), and possible theories have recently been reviewed by Tavares (1966).

In the carcinoma of the colon (Patient No. 4), the high incidence of cells with triple sex chromatin was noteworthy ; however, one region of this tumour differed from the remainder in that the cells had only two sex chromatin bodies. There was thus evidence from the sex chromatin findings of the existence of two stem-lines in this tumour, as well as in the carcinoma of the breast mentioned above ; whether these cell-lines arose independently, or whether one line arose from the other during the course of development of the tumours is of course uncertain.

Taken as a whole the sex chromatin findings suggest that replication of the allocyclic X in tumours is a more or less random event occurring more frequently, though not solely, in tumours with a high chromosome number. Three out of the four tumours with triple sex chromatin were near-tetraploid, while the fourth was hyperdiploid ; it was previously found from data on DNA content (Atkin, 1964) that most of the tumours with double sex chromatin were near-tetraploid. It is not clear however why most near- (often hypo-) tetraploid tumours of females are chromatin-negative (Atkin, 1964). Possibly in these tumours loss of the allocyclic X (probably in company with other chromosomes) first resulted in a hypodiploid condition and, subsequently, a complete chromosomal doubling resulted in hypotetraploidy. Alternatively, it is possible that absence of sex chromatin does not necessarily imply absence of the allocyclic X, and that some other mechanism may be involved in the loss of ability to form sex chromatin.

It has been noted elsewhere (Atkin, 1964) that there is a relationship between sex chromatin content and prognosis in carcinoma of the cervix and corpus uteri : single-sex chromatin cervix tumours have a relatively poor prognosis while, on the other hand, single-sex chromatin corpus tumours have a relatively favourable prognosis. This may be a consequence of the relationship between sex chromatin and ploidy-level (near-diploid tumours usually having single sex chromatin, and near-tetraploid tumours double or absent sex chromatin (Atkin, 1964)) rather than an effect of sex chromatin *per se* ; recent data on uterine tumours have reaffirmed an influence of ploidy-level on prognosis in uterine tumours (Atkin, 1966). Similarly, the apparent relationship between presence or absence of sex chromatin in breast tumours and their response to hormone therapy (see review by Tavares, 1966) might be an expression of a relationship between sex chromatin content and ploidy-level.

### SUMMARY

Four tumours out of a series of 732 malignant tumours of females were characterised by the presence of triple sex chromatin (i.e. the most frequently-occurring number of sex chromatin bodies per nucleus was 3). Two of the tumours had been reported previously ; the two new cases are described in detail. In one, a carcinoma of the colon, triple sex chromatin was identified in 77% of suitable nuclei (64% of all nuclei) in squash preparations. Sections taken from 5 different tumour regions showed triple sex chromatin consistently in 4, but about a third of the tumour on the fifth section had only two bodies per nucleus. Of the other tumours, 58% had a single sex chromatin body, 10% had double sex chromatin, and 31% were chromatin-negative. On the other hand, each of 324 malignant non-teratomatous tumours from male patients was chromatin-negative. The findings are discussed in the light of the possibility that deviations from the single sex chromatin pattern in tumours of females bear a direct relation to changes (absence, duplication or triplication) involving the allocyclic X chromosome.

The author wishes to thank Mrs. M. Mason for technical assistance, and Mrs. P. Oliver and Mrs. B. Langdon for secretarial services. The leucocyte culture on Patient No. 4 was performed by Mrs. S. Wilson. This work was supported by a grant from the British Empire Cancer Campaign for Research.

### REFERENCES

ATKIN, N. B.—(1958) 'Observations on sex chromatin in human tumours'. *In* 'Symposium on Nuclear Sex,' edited by D. Robertson Smith and W. M. Davidson. London (Heinemann), p. 168.—(1960) *Acta Un. int. Cancr.*, **16**, 41.—(1964) *Wien. klin. Wschr.*, **49**, 859.—(1966) *Proc. R. Soc. Med.* **59**, 979.

ATKIN, N. B. AND BAKER, M. C.—(1966) *J. natn. Cancer Inst.*, **36**, 539.

ATKIN, N. B. AND ROSS, A. J.—(1960) *Rep. Br. Emp. Cancer Campn*, **38**, 230.

GALTON, M., BENIRSCHKE, K., BAKER, M. C. AND ATKIN, N. B.—(1966) *Cytogenetics*, **5**, 261.

LYON, M.—(1961) *Nature, Lond.*, **190**, 372.

TAVARES, A. S.—(1966) 'Sex chromatin in tumours'. *In* 'The Sex Chromatin', edited by K. L. Moore. Philadelphia (W. B. Saunders), p. 405.